# Clinical application of the supraorbital key-hole approach to the treatment of unilateral-dominant bilateral frontal contusions

**DOI:** 10.18632/oncotarget.15983

**Published:** 2017-03-07

**Authors:** Shuguang Zhang, Chunfa Qian, Guan Sun, Xiaoliang Li

**Affiliations:** ^1^ Department of Neurosurgery, The First People’s Hospital of Kunshan Affiliated with Jiangsu University, Suzhou, P. R. China; ^2^ Department of Neurosurgery, Affiliated Nanjing Brain Hospital, Nanjing Medical University, Nanjing, P. R. China; ^3^ Department of Neurosurgery, Fourth Affiliated Yancheng Hospital of Nantong University, Yancheng, P. R. China

**Keywords:** key-hole approach, bilateral frontal contusion, craniotomy

## Abstract

We compared the surgical efficacy of the supraorbital key-hole approach (SKA) to conventional unilateral frontotemporal craniotomy (UFTC) for the treatment of patients with unilateral-dominant bilateral frontal contusions (BFCs). A retrospective analysis of 62 patients with unilateral-dominant BFCs who underwent surgery at our institute between 2014 and 2017 was performed. There were 26 patients who underwent SKA (group A) and 36 who underwent UFTC (group B). Postoperative computed tomography scans showed satisfactory evacuation of the frontal cerebral contusions in both groups (*p* > 0.05). There was less intraoperative blood loss in group A than group B (17.1 ± 4.55 *vs*. 67.6 ± 10.28 mL, *p* < 0.05). The operative time was also shorter in group A (82.7 ± 13.73 vs. 132.4 ± 9.17 min, *p* < 0.05). Postoperative bleeding occurred in three cases in group A and in only one case in group B (*p* > 0.05). The average length of hospitalization was shorter in group A than group B (7.3 ± 1.09 *vs*. 12.9 ± 1.71 days, *p* < 0.05). No differences in the Glasgow Outcome Scale were observed between the two groups after 6 months of follow-up (*p* > 0.05). Thus, compared to UFTC, SKA is associated with shorter operation times and less trauma to the surrounding brain tissue.

## INTRODUCTION

A bilateral frontal contusion (BFC) is a common traumatic brain injury in clinical practice [1]. Because the injury site is close to the cerebral midline, frontal cerebral contusions can easily cause central herniations that significantly impact patient prognosis [2]. Bilateral frontotemporal craniotomy may effectively evacuate the hematoma and achieve decompression, but the approach has drawbacks such as a longer operative time, intraoperative blood loss, and trauma that may result in complications. Microscope-assisted surgical techniques, such as frontotemporal craniotomy via a small bone window combined with a falx incision, have been shown to greatly reduce brain tissue injury and improve the prognosis of BFC patients [3]. However, patients with unilateral-dominant BFCs have traditionally been treated with unilateral frontotemporal craniotomy (UFTC) via a small bone window to evacuate contused brain tissue and intracerebral hematomas. Recently, we found that the supraorbital key-hole approach (SKA) was also effective for the treatment of patients with unilateral-dominant BFCs. In this study, we retrospectively analyzed 62 patients with unilateral-dominant BFCs and compared the treatment efficacy of SKA to conventional UFTC. We found that the SKA had the following advantages: less trauma, shorter operative times, faster postoperative recovery, and fewer complications.

## RESULTS

### No significant differences were observed in routine monitoring indexes prior to treatment with the SKA or conventional UFTC

We first assessed the clinical features of patients treated with the SKA (group A) or conventional UFTC (group B). No significant differences between the two groups were observed in routine monitoring indexes including the Glasgow Coma Scale (GCS), age, sex, temperature, heart rate, respiration rate, blood pressure, or volume of the unilateral-dominant BFC before surgery (Table 1).

**Table 1:** Clinical features of the patients who were treated using the SKA or conventional UFTC

Observation Index	Group A	Group B	p
Preoperative GCS	11.1 ± 1.38	10.9 ± 1.41	0.603
N			
Age, y	40.8 ± 13.3	46.4 ± 13.1	0.105
Sex			
Male	19	23	
Female	7	13	0.445
Temperature (°C)	36.6 ± 0.50	36.8 ± 0.55	0.129
Heart rate	81.1 ± 20.95	78.2 ± 12.46	0.490
Respiration rate	22.1 ± 3.95	23.4 ± 3.50	0.209
Blood pressure (Systolic pressure, mmHg)	125.7 ± 20.19	134.0 ± 19.90	0.113
Volume of cerebral contusion (unilateral-dominant, mL)	32.6 ± 4.00	32.06 ± 3.67	0.603

### Comparison of perioperative parameters between groups A and B

We performed a statistical analysis of various perioperative parameters in groups A and B (Table 2). We found that the removal rate of the contused brain tissue and hematomas was greater than 90% in 23 of 26 patients in group A, and in 35 of 36 patients in group B (p > 0.05). There was less intraoperative blood loss in group A than in group B (17.1 ± 4.55 vs. 67.6 ± 10.28 mL, p < 0.05). Additionally, the operative time was shorter in group A than in group B (82.7 ± 13.73 vs. 132.4 ± 9.17 min, p < 0.05). Head computed tomography (CT) was repeated 1-3 days after surgery. In group A, re-bleeding in the surgical field was identified in 3 of 26 patients, which could be managed with conservative treatments such as hemostasis and dehydration therapy. Re-bleeding was observed in one of 36 patients in group B. The re-bleeding rates were 11.5% and 2.8% in groups A and B, respectively (p > 0.05). During the period of hospitalization, no high fevers (> 38.5°C) were reported in either group. However, low fevers (< 38.2°C) were reported in five patients in group A and six in group B. Stiff neck was not noted in either group. All symptoms subsided after treatment. Low fevers were likely postoperative, absorption-induced, and intracranial infection was ruled out. These results suggested that the incidence of intracranial infection did not differ between the two groups (p > 0.05). Finally, the length of hospital stay was significantly shorter in group A than in group B (7.3 ± 1.09 vs. 12.9 ± 1.71 days, p < 0.05).

**Table 2:** Comparison of various perioperative parameters between groups A and B

Observation Index	Group A	Group B	p
**Hematoma removal rate > 90% (n)**	23/26	35/36	0.300
**Intraoperative blood loss (mL)**	17.1 ± 4.55	67.6 ± 10.28	0.000
**Operative time, min**	82.7 ± 13.73	132.4 ± 9.17	0.000
**Bleeding recurrence rate in the surgical field (n)**	3/26	1/36	0.300
**Intracranial infection (n)**	0/26	0/36	0.000
**Length of hospitalization (d)**	7.3 ± 1.09	12.9 ± 1.71	0.000

## DISCUSSION

BFCs are predominantly associated with deceleration injuries such car accidents and falls from high places in which the frontal brain tissue slips and strikes the rough bone crest of the skull bottom [4]. The injured sites are in the anterior and bottom portions of the frontal lobe. CT scans may reveal scattered hemorrhages in the brains of some patients in the early stages. Most patients with BFCs present with relatively mild degrees of acute consciousness disorders, are stable after treatment, and ultimately recover [5]. However, some patients may present with extensive BFCs and intracerebral hematomas with disease progression. The lateral-anterior frontal lobe is attached to the frontotemporal bone surface of the skull base, and the medial frontal lobe is adjacent to the falx cerebri. Therefore, a frontal cerebral contusion or hematoma may lead to increased volume and compression of the hypothalamus or brain stem [6]. In patients with severe contusions, central herniations may be present due to compression and shifting of the deep midline structures of the hemisphere [2]. Once central herniation is present, the patient may enter a coma. Thus, this condition must be recognized and treated in a timely manner to prevent poor patient outcomes.

Because central herniations may occur suddenly, early surgical treatment is critical to reduce the mortality rate of patients in the early stages when central hernia is not present. Therefore, we recommend the use of a less strict definition of the indications for surgery in order to improve the treatment success rate in patients with BFCs. Traditional guidelines suggest that surgical treatment should be performed immediately if the following signs are present in patients with traumatic brain injuries: contused volume of the frontal, temporal, or parietal lobes > 20 mL and a midline shift > 5 mm with basal cistern compression [7]. We suggested that surgical treatment is indicated if (1) conscious patients experience mild disturbances of consciousness and restlessness, miosis, slow pupillary light reflex, increased blood pressure, faster pulse and respiration, and increased muscle tone; (2) dynamic CT monitoring shows a trend toward increased primary and scattered contusion spots or significantly expanded edema and compression of the frontal horn of the lateral ventricle; and (3) significant enlargement of unilateral contusions, or intracerebral hematoma and a > 5 mm shift of the anterior midline to the contralateral side, which could be accompanied by decreased consciousness.

The guidelines for the management of traumatic brain injuries in both China and in the United States recommend the use of bifrontal decompressive craniotomy via coronal incision in patients with BFCs and/or diffuse brain swelling [8]. Polin et al. demonstrated that bifrontal decompressive craniotomy was effective for the treatment of malignant brain edema [9]. Some studies have shown that bifrontal decompressive craniotomy can effectively reduce intracranial pressure (ICP) and increase cerebral perfusion pressure (CPP) in patients with refractory intracranial hypertension and bilateral diffuse brain swelling [10]. Unlike other types of craniotomy, bifrontal decompressive craniotomy was designed to directly relieve ICP from the anterior cranial cavity [11, 12]. This method can effectively reduce ICP, stabilize intracranial structures, reduce secondary brain damage, and lower the risk of recurrence of central hernia caused by extensive, intractable cerebral edema. However, with the development of microscope-assisted surgical techniques, some clinicians have utilized microscope-assisted unilateral craniotomy and falx incision for the treatment of BFCs [3]. Previous studies have shown that unilateral craniotomy and microscope-assisted surgical techniques can also be used to remove contralateral frontal hematomas and to completely preserve the contralateral frontotemporal structures outside the brain. These techniques have several advantages including a shorter operative time, less blood loss, and faster postoperative recovery [13].

We believe that conventional microscope-assisted UFTC for the treatment of patients with unilateral-dominant BFCs has several disadvantages including longer operative times and significant tissue trauma. Because unilateral-dominant BFCs differ significantly from diffuse BFCs, we suggest performing evacuations of any cerebral contusions on the dominant side of the injury and treating the side with the mild injury using conservative modalities. Based on this concept, we proposed a novel approach (i.e. unilateral evacuation of the frontal cerebral contusion and hematoma using the SKA). The SKA is based on the concept of minimally invasive surgery. It has clear advantages including minimal tissue injury and quick postoperative recovery times [14]. This approach is frequently used by neurosurgeons to treat small lesions such as tumors located in the anterior cranial fossa [15]. However, the application of the SKA to the treatment of BFCs has not been previously reported because of concerns including postoperative edema and re-bleeding.

Our results demonstrate that the treatment efficacy of evacuation of frontal cerebral contusions and hematomas using the SKA did not significantly differ from that of conventional frontotemporal craniotomy. Additionally, the SKA resulted in shorter operative times, less intraoperative blood loss, and shorter average hospital stay lengths compared to conventional UFTC. No significant differences in the incidence rate of postoperative bleeding and intracranial infection were observed between patients treated with the SKA or UFTC. Importantly, there was no difference in the Glasgow Outcome Scale (GOS) scores after the 6-month follow-up. These results suggest that SKA does not compromise patient prognosis.

The choice to use the SKA should be based on the BFC and physician experience. Brain edema is the most common problem that develops during surgery for traumatic brain injuries. Thus, the appropriate selection of patients for the SKA and physician experience are critical. We generally selected patients with approximately 30 mL dominant lateral brain contusions and only slight contusions on the contralateral side. These patients did not have other areas of severe brain damage, swelling, or subdural hematomas. In addition, the basal cisterns was still present. Our data suggest that the SKA may be a feasible, safe, and effective treatment for patients with unilateral-dominant BFCs.

## MATERIALS AND METHODS

### Data collection

A total of 62 patients with unilateral-dominant BFCs who were treated at our institute between January 2015 and November 2016 were selected for this study. There were 35 male and 27 female patients, with an average age of 44.0 ± 13.37 years (range: 19-70 years). All patients were directly struck on the occipital region. There were 26 patients who underwent evacuation of frontal cerebral contusions using the SKA (microscope-assisted) and 36 patients who underwent UFTC. Preoperative sex, age, and GCS score did not significantly differ between the two groups. This study was approved by the Hospital Ethics Committee, and the patients and their family members provided informed consent.

### Indications for emergency surgery

Patients with mild brain injuries (GCS score: 13-15) were treated conservatively on admission (i.e. consciousness was closely monitored and patients underwent dynamic head CT). Treatment included dehydration therapy, sedation, nerve protection, anti-infectives, and treatments to prevent complications. Surgery was indicated if patients did not respond to treatment and any of the following signs were observed: progressive loss of consciousness, decreased GCS score, head CT showing unilateral frontal contusions with exacerbated brain edema, obvious compression of the frontal horn of the lateral ventricle, significant midline shift to the opposite side, a significant mass effect.

### Exclusion criteria

Patients with the following conditions were excluded from the study: (1) concomitant primary brain stem injury; (2) concomitant brain contusion or an intracerebral hematoma other than in the frontal lobe; (3) concomitant heart, liver, lung, and/or kidney dysfunction; (4) concomitant hemorrhagic shock, severe coagulation abnormalities, and/or multiple organ failure; and (5) history of brain tumors and/or cerebral infarction.

### Surgical procedures

#### Unilateral evacuation of frontal cerebral contusions using the SKA (group A)

**Figure 1:**
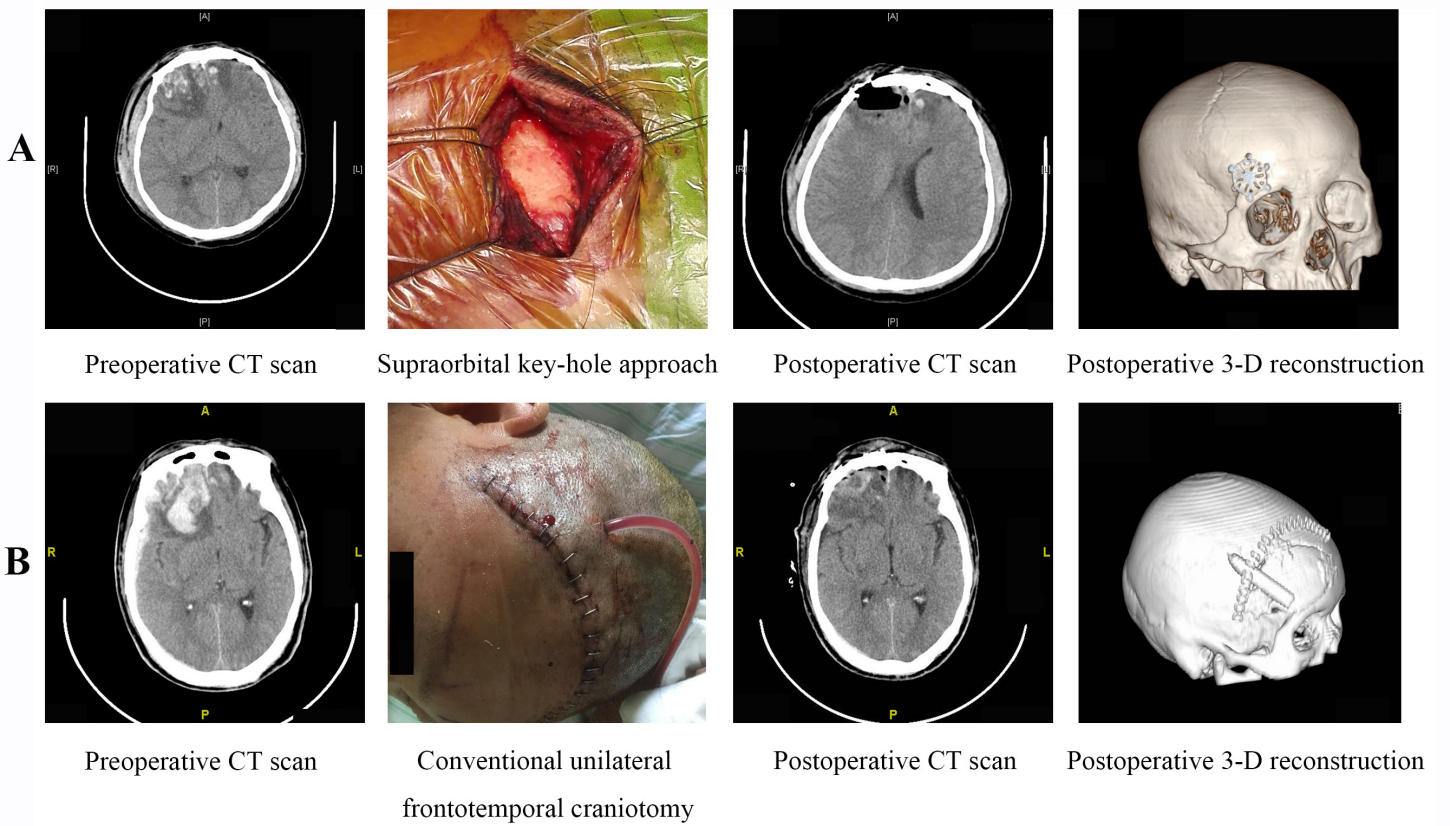
Surgical Procedure **A**. The SKA and **B**. UFTC for the treatment of unilateral-dominant BFCs.

Patients were placed in the supine position and the head secured on a multi-functional head frame. The head was positioned according to the location of the lesion and was tilted 10-25 degrees to the contralateral side and bent 10-15 degrees posteriorly. In this position, the frontal base could naturally separate from the skull base under gravity to reduce intraoperative retraction of the brain. A site lateral to the superciliary arch was selected to access the skull cavity. A 3-4 cm skin incision was made along the superciliary arch until the periosteum was reached. The subcutaneous tissues were separated and the edges of the incision suspended through sutures. A bone drill was used to drill a 2-3 cm hole at the site lateral to the superciliary arch. A curved incision was made in the dura mater, with the base toward the eyeball. Contused brain tissue and hematomas were visualized under a microscope and carefully evacuated. Larger hematomas, which had been previously divided using micro-scissors, were removed piece by piece rather than as a whole. Hemostatic gauze was used to fill the residual cavity after removal of the hematomas and contused brain tissue. The dura mater was tightly closed after complete hemostasis. An artificial plate was used to cover the hole in the bone and the cranial cavity closed. No drainage tube was placed in the surgical field. The surgical procedure is shown in Figure 1A.

#### Evacuation of frontal cerebral contusions using conventional UFTC (group B)

Patients were placed in the supine position under general anesthesia. A conventional frontotemporal incision was made and the craniotomy performed on the side of the more severe brain injury. The skin flap was then retracted anteriorly-inferiorly and the galea aponeurotica dissected until the proximal ends of the superciliary arches were reached. On the side of the more severe brain contusion, four holes were drilled using a bone drill to form a 3 × 3 cm window. The dura mater was suspended and incised in a curved fashion. The base of the curved incision was oriented toward the eyeball. The contused brain tissue and any hematomas were visualized under a microscope and carefully removed. Strict hemostasis was performed in the surgical field. A drainage tube was inserted after surgery. After closure of the dura mater, either a routine closure or decompression closure of the cranial cavity was performed depending on the brain beat pulsation, edema, and the effectiveness of the intra-decompression (Figure 1B).

### Observation parameters

The following patient parameters were recorded after craniotomy: (1) the removal rate of the contused frontal brain tissue and hematoma ≥ 90%; (2) the mean operative time; (3) intraoperative blood loss; (4) the incidence rate of re-bleeding in the surgical field; (5) the incidence rate of intracranial infection; (6) the average length of hospital stay; and (7) the GOS scores at the 6-month follow-up, in which a score of 1 indicated death, a score of 2 indicated patient survival, a score of 3 indicated severe disability, a score of 4 indicated moderate disability, and a score of 5 indicated mild or no disability.

### Statistical analysis

The data were analyzed using SPSS version 15.0 (SPSS Inc., Chicago, Illinois, USA). Quantitative variables including age and temperature were expressed as the mean ± standard deviation (SD). We used χ2 or Fisher's analyses to evaluate the relationships between categorical variables and t-tests for continuous variables. A p < 0.05 was considered statistically significant.

## References

[1] Gupta DK, Singla R, Kale SS, Sharma BS (2016). Intracerebral hypoglycemia and its clinical relevance as a prognostic indicator in severe traumatic brain injury: A cerebral microdialysis study from India. Neurol India.

[2] Wu H, Yang SF, Qiu YM, Dai J, Li SQ, Zhang XH, Miao YF (2014). The diagnosis and surgical treatment of central brain herniations caused by traumatic bifrontal contusions. J Craniofac Surg.

[3] Gao L, Wu X, Hu J, Jin Y, Han X, Wu X, Mao Y, Zhou L (2013). Intensive management and prognosis of 127 cases with traumatic bilateral frontal contusions. World Neurosurg.

[4] Ji-Rong D, Qin-Yi X, Xue-Jian C, Biao W, Yu-Hai W, Zhong-Hua S, Bin L, Sang C, Jian-Qing H, Xu H (2012). Endoscopy-assisted cerebral falx incision via unilateral approach in treatment of dissymmetric bilateral frontal contusion. J Craniofac Surg.

[5] Dong JR, Cai XJ, Wang B, Wang YH, Shi ZH, Liu B, Cai S, Xu QY (2010). Intracranial pressure monitoring for special patterns of frontal lobe contusions. Chin J Traumatol.

[6] Statham PF, Johnston RA, Macpherson P (1989). Delayed deterioration in patients with traumatic frontal contusions. J Neurol Neurosurg Psychiatry.

[7] Adams H, Kolias AG, Hutchinson PJ (2016). The Role of Surgical Intervention in Traumatic Brain Injury. Neurosurg Clin N Am.

[8] Magnone S, Allegri A, Belotti E, Castelli CC, Ceresoli M, Coccolini F, Manfredi R, Merli C, Palamara F, Piazzalunga D, Valetti TM, Ansaloni L (2016). Impact of ATLS guidelines, trauma team introduction, and 24-hour mortality due to severe trauma in a busy, metropolitan Italian hospital: A case control study. Ulus Travma Acil Cerrahi Derg.

[9] Polin RS, Shaffrey ME, Bogaev CA, Tisdale N, Germanson T, Bocchicchio B, Jane JA (1997). Decompressive bifrontal craniectomy in the treatment of severe refractory posttraumatic cerebral edema. Neurosurgery.

[10] Elwatidy S (2006). Bifrontal decompressive craniotomy for malignant brain edema. Saudi Med J.

[11] Pereira WC, Neves VJ, Rodrigues Y (1977). [Bifrontal decompressive craniotomy as the treatment for severe cerebral edema]. [Article in Portuguese]. Arq Neuropsiquiatr.

[12] Kjellberg RN, Prieto A (1971). Bifrontal decompressive craniotomy for massive cerebral edema. J Neurosurg.

[13] Ucar T, Akyuz M, Kazan S, Tuncer R (2005). Role of decompressive surgery in the management of severe head injuries: prognostic factors and patient selection. J Neurotrauma.

[14] Reisch R, Stadie A, Kockro R, Gawish I, Schwandt E, Hopf N (2009). The minimally invasive supraorbital subfrontal key-hole approach for surgical treatment of temporomesial lesions of the dominant hemisphere. Minim Invasive Neurosurg.

[15] Iacoangeli M, Nocchi N, Nasi D, Di Rienzo A, Dobran M, Gladi M, Colasanti R, Alvaro L, Polonara G, Scerrati M (2016). Minimally Invasive Supraorbital Key-hole Approach for the Treatment of Anterior Cranial Fossa Meningiomas. Neurol Med Chir (Tokyo).

